# Two-Dimensional Matrix Algorithm Using Detrended Fluctuation Analysis to Distinguish Burkitt and Diffuse Large B-Cell Lymphoma

**DOI:** 10.1155/2012/947191

**Published:** 2012-12-29

**Authors:** Rong-Guan Yeh, Chung-Wu Lin, Maysam F. Abbod, Jiann-Shing Shieh

**Affiliations:** ^1^Department of Mechanical Engineering, Yuan Ze University, 135 Yuan-Tung Road, Chungli 32003, Taiwan; ^2^Department of Pathology, National Taiwan University Hospital, Taipei 100, Taiwan; ^3^School of Engineering and Design, Brunel University, London UB8 3PH, UK; ^4^Center for Dynamical Biomarkers and Translational Medicine, National Central University, Chungli 32001, Taiwan

## Abstract

A detrended fluctuation analysis (DFA) method is applied to image analysis. The 2-dimensional (2D) DFA algorithms is proposed for recharacterizing images of lymph sections. Due to Burkitt lymphoma (BL) and diffuse large B-cell lymphoma (DLBCL), there is a significant different 5-year survival rates after multiagent chemotherapy. Therefore, distinguishing the difference between BL and DLBCL is very important. In this study, eighteen BL images were classified as group A, which have one to five cytogenetic changes. Ten BL images were classified as group B, which have more than five cytogenetic changes. Both groups A and B BLs are aggressive lymphomas, which grow very fast and require more intensive chemotherapy. Finally, ten DLBCL images were classified as group C. The short-term correlation exponent **α**1 values of DFA of groups A, B, and C were 0.370 ± 0.033, 0.382 ± 0.022, and 0.435 ± 0.053, respectively. It was found that **α**1 value of BL image was significantly lower (*P* < 0.05) than DLBCL. However, there is no difference between the groups A and B BLs. Hence, it can be concluded that **α**1 value based on DFA statistics concept can clearly distinguish BL and DLBCL image.

## 1. Introduction

Natural phenomena almost are random, nonlinear, nonstationary, disordered, and uncertain systems. It is difficult to follow the traditional Newtonian rules to be completely forecast or control. Therefore, opening up fractal methods to investigate complex, rough, fragment shape, and noninteger dimension naturally objects (trees, coastlines, clouds, and mountains, etc.) is necessity. In the 1960s, the mathematician Mandelbrot had been indicated the fractal objects whose complex geometry cannot be characterized by an integral dimension [1]. This phenomenon is often expressed by spatial or time-domain statistical scaling laws and is mainly characterized by the power-law behavior of real-world physical systems. It represents fractal applies to objects in space or fluctuations in time that possess a form of self-similarity [2]. The object has self-similarity means; the variant of object expressed same qualitatively, irrespective reduction or magnification of the object. Self-similarity is one way to calculate fractal dimension. For example, one can subdivide a line segment into *m* self-similar intervals, each with the same length, and each of which can be magnified by a factor of *n* to yield the original segment [3]. Due to the fractal geometry having an approximately copy of the whole, the fractal dimension is consistent over a wide range of scales, which is known as scale invariance [4]. This property provided a useful measurement of complexity object.

DFA method was developed from a modified root mean square analysis of a random walk to exclude the local trend induced by characteristic time scales from the fluctuations of the multicomponent systems and get a long-range correlation [5–7]. It was originally a method to measure scale invariant behavior developed by Peng et al. [6] that evaluated trends of all sizes in the presence or absence of fractal correlation properties of time series data [8, 9]. This method has been applied to heart rate dynamics such as autonomic nervous system [10], congestive heart failure [8], dilated cardiomyopathy [11], ventricular fibrillation [12], and other physiological nonstationary time series systems (DNA sequences [13], neuron spiking [14, 15], human gait analysis [16], electroencephalogram (EEG) in sleep [17–20], stock returns [21], periodic trends [22], estimating dependence [23], etc.). Experience has shown that monodimensional detrended fluctuation analysis (DFA) used in the scaling analysis of fractal time series is accurate and easy to implement regardless in long-term and short-term time scale series [24, 25]. In recent years, there are some modified DFA method researches that are proposed such as generalized the monodimensional DFA and multifractal detrended fluctuation analysis (MFDFA) to higher-dimensional versions and derived multifractal detrended cross-correlation analysis method to investigate the multifractal behaviors in the power-law cross correlations between two time series or higher-dimensional quantities recorded [26–28]. The multifractal detrended cross-correlation analysis based on DFA (MF-X-DFA) [27] is actually a multifractal generalization of the detrended cross-correlation analysis (DCCA) [29], which has other variants such as the multifractal detrended cross-correlation analysis based on DMA (MF-X-DMA) [30]. Those study results validated well for distinguishing fractal/multifractal properties of synthetic surfaces (including fractional Brownian and multifractal surfaces), one/two-dimensional cross correlation of two financial time series, and linear/nonlinear correlation analysis of traffic time series (to find the cross correlation of traffic flow and volume data).

**Figure 1:**
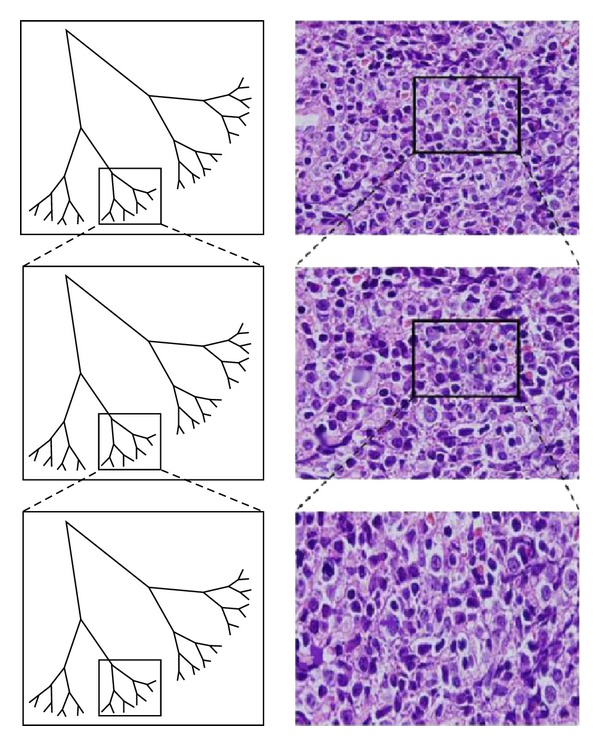
Fake tree (left hand side) and real lymphoma cell (right hand side) showed the self-similarity characteristics of pattern repeated in different zoom scales.

Although there are many varieties of malignant lymphomas, one of them is aggressive B-cell lymphoma. Diffuse large B-cell lymphoma (DLBCL) is the largest category of aggressive B-cell lymphomas. Less than 50% of patients can be cured by combination chemotherapy [31]. DLBCL has two important subgroups, which are germinal center B-cell-like (GCB) and activated B-cell-like (ABC) lymphoma. In medicine, cDNA microarrays method can successfully use to distinguish GCB and ABC DLBCL. The advantage of distinguish GCB and ABC DLBCL subgroups has significantly different 5-year survival rates after multiagent chemotherapy (GCB over 60%) [32, 33]. A similar situation exists between Burkitt lymphoma (BL) and DLBCL. Both lymphomas were all classified as aggressive B-cell non-Hodgkin's lymphoma in the World Health Organization [34]. Therefore, how to distinguish the difference between BL and DLBCL is a challenge, as the two diseases require different treatment and have different cure rate. Existing diagnosis and classification between BL and DLBCL evaluated their morphologic, immunophenotypic, and cytogenetic features and clinical outcomes [35, 36]. Recently, the Cui et al. study [37] had successful applied nonmedical methods (i.e., statistical and engineering methods, linguistic analysis, and ensembled artificial neural networks) to classify two types of GCB and ABC DLBCL. Because fractal temporal process may generate fluctuations on different area scales that are statistically self-similarity [38], therefore, the same concept of fractal temporal process and the statistically self-similarity of cell image are used as shown in Figure 1 because the lymphoma cells exist big and small cells at the same time which can easily display statistical self-similarity characteristics. In this paper, a nonmedical method/two-dimensional (2D) algorithms of DFA has been proposed based on the original design method concepts. The proposed method was used to recharacterize the images of lymph sections. It is anticipated that 2D DFA could be helpful to distinguish BL and DLBCL section images.

## 2. Material and Methods

### 2.1. Material

**Figure 2:**
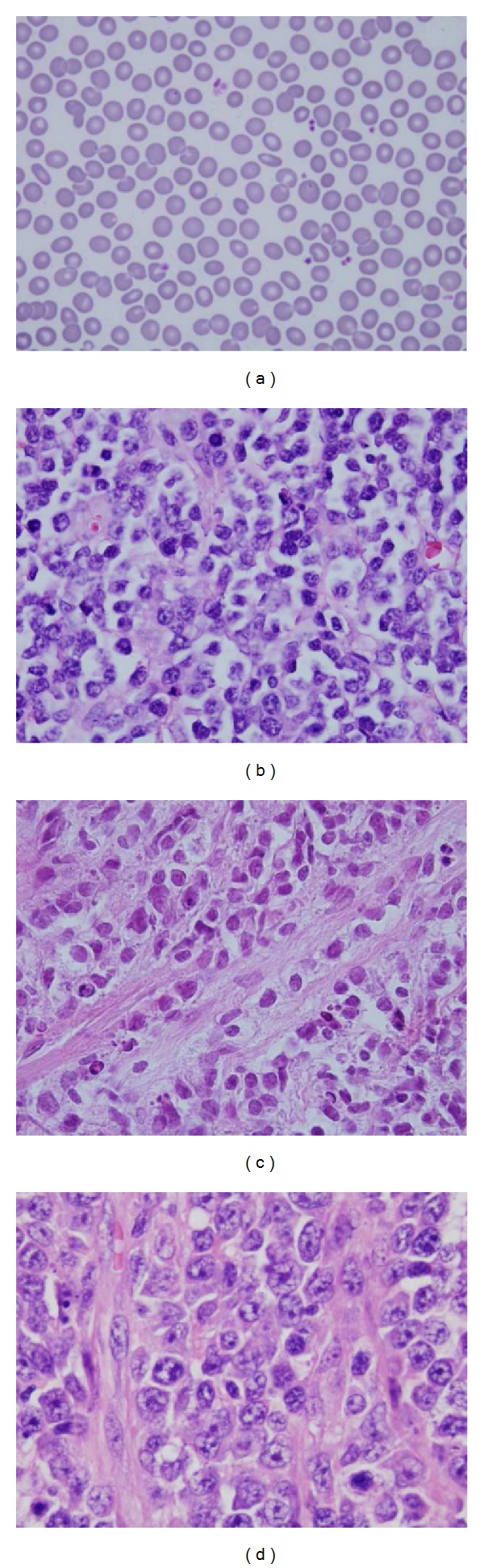
Healthy cell images, BL and DLBCL. (a) Healthy cell image, (b) BL image (one to five cytogenetic changes), (c) BL image (more than five cytogenetic changes), and (d) DLBCL image, which image is 4080 × 3072 pixels.

A total of 38 lymph section images cataloged into 3 lymphoma groups were used in the classification as shown in Table 1. Eighteen BL images were classified as group A, which have one to five cytogenetic changes. Ten BL images were classified as group B, which have more than five cytogenetic changes. Both group A and B BLs are high grade aggressive lymphomas, which grow very fast and require more intensive chemotherapy. Finally, ten DLBCL images were grouped as C. Some images of healthy cell, BL and DLBCL (4080 × 3072 pixels), are shown in Figure 2. 

### 2.2. Two-Dimensional Analysis Algorithm of DFA

The algorithm of monodimensional DFA method described in [6] quantifies fractal-like correlation properties by calculating the scaling property of the root-mean-square fluctuation of the integrated and detrended time series data. To illustrate the DFA algorithm, a time series signal (with *N* samples) is used and analyzed as in the following equations:

(1) $$\begin{array}{c} y\left(k\right)=\sum_{i=1}^{k}\left[B\left(i\right)-\bar{B}\right], \end{array}$$

where *B*(*i*) is the *i*th sample of a signal; $\bar{B}$ is the average of overall signal; and *y*(*k*) is the value of the *k*th sample of the integrated time series. Then, the fluctuation of integrated and detrended time series for a given window with scale of *n* is calculated by

(2) $$\begin{array}{c} F\left(n\right)=\sqrt{\frac{1}{N}\sum_{k=1}^{N}{\left[y\left(k\right)-y_{n}\left(k\right)\right]}^{2}}, \end{array}$$

where *F*(*n*) is the fluctuation of an integrated time series for a given window with scale of *n*, and *y*_*n*_(*k*) is the *k*th point on the trend derived using a predetermined window with scale of *n*. A straight line of log⁡⁡(*F*(*n*)) versus log⁡⁡(*n*) plot indicates the presence of power law (fractal) correlation between scales and the fluctuations of the detrended time series. The slope of log-log plot is defined the DFA scaling exponent **α**. For the **α** exponent, Peng et al. indicate it as an indicator that describes the “roughness” of the original time series: the larger the value of **α**, the smoother the time series [6].

As the application of DFA in image analysis, two-dimensional algorithm of DFA should be refined in both integration and detrending processes. Considering the integration in both dimensions, the formula of integration should be as the following steps.

**Step 1**

The color image should be gray-scale processing at first. Consider a self-similar surface, which is denoted by a two-dimensional array *B*(*i*, *j*), where *i* = 1, 2,…, *M* and *j* = 1, 2,…, *N*. Content of *B*(*i*, *j*) is the pixel value of surface image. Like monodimensional DFA method, the first integrated equation is as follows:

(3) $$\begin{array}{c} y\left(m,n\right)=\sum_{i=1}^{m}\left[B\left(i,n\right)-{\bar{B}}_{nc}\right]+\sum_{j=1}^{n}\left[B\left(m,j\right)-{\bar{B}}_{mr}\right] \end{array}$$

for 1 ≤ *m* ≤ *M*, 1 ≤ *n* ≤ *N*, where *y*(*m*, *n*) is the value of the pixel (*m*, *n*) on the integrated image, *B*(*i*, *n*) is the *i*th pixel on the *n*th column, ${\bar{B}}_{nc}$ is the average of the *n*th column, *B*(*m*, *j*) is the *j*th pixel on the *m*th row, and ${\bar{B}}_{mr}$ is the average of the *m*th row.

**Step 2**

The surface is partitioned into *M*_*s*_ × *N*_*s*_ disjoint square segments of the same size *s* × *s*, where *M*_*s*_ = [*M*/*s*], *N*_*s*_ = [*N*/*s*], and 4 ≤ *s*≤min (*M*/4,  *N*/4). *y*(*m*, *n*) can be denoted by *y*_*k*,*l*_, segments such that *y*_*k*,*l*_(*o*, *p*) = *y*(*k*_1_ + *o*, *l*_1_ + *p*) for 1 ≤ *o*, *p* ≤ *s*, 1 ≤ *k* ≤ *M*_*s*_, and 1 ≤ *l* ≤ *N*_*s*_, where *k*_1_ = (*k* − 1)*s* and *l*_1_ = (*l* − 1)*s*.

**Step 3**

Use least square method to calculate the trend of matrix *y*_*k*,*l*_(*s*, *s*) expressed as ${\hat{y}}_{k,l}(s,s)$. The trend matrix ${\hat{y}}_{k,l}(s,s)$ is the mathematical model of linear regression. The trend decomposes into row and column, two directions.

**Step 4**

The mean squared error (MSE) represents the fluctuation of an integrated and detrended segment image with scale of *s* × *s* as

(4) $$\begin{array}{c} E_{k,l}=\frac{1}{s^{2}}\sum_{o=1}^{S}\sum_{p=1}^{S}{\left[y_{k,l}\left(o,p\right)-{\hat{y}}_{k,l}\left(s,s\right)\right]}^{2} \end{array}$$

for *E*_*k*,*l*_ value expected to be minimized.

**Step 5**

The overall detrended fluctuation is calculated by averaging overall the segments, that is

(5) $$\begin{array}{c} F^{2}\left(s\right)=\frac{1}{M_{S}N_{S}}\sum_{k=1}^{M_{S}}\sum_{l=1}^{N_{S}}E_{k,l}. \end{array}$$

Substituting (4) into (5) to get (6)

(6) $$\begin{array}{c} F^{2}\left(s\right)=\frac{1}{M_{S}N_{S}}\sum_{k=1}^{M_{S}}\sum_{l=1}^{N_{S}}\left\{\frac{1}{s^{2}}\sum_{o=1}^{S}\sum_{p=1}^{S}{\left[y_{k,l}\left(o,p\right)-{\hat{y}}_{k,l}\left(s,s\right)\right]}^{2}\right\}. \end{array}$$

Then

(7) $$\begin{array}{c} F\left(s\right)=\sqrt{\frac{1}{M_{S}N_{S}}\sum_{k=1}^{M_{S}}\sum_{l=1}^{N_{S}}\left\{\frac{1}{s^{2}}\sum_{o=1}^{S}\sum_{p=1}^{S}{\left[y_{k,l}\left(o,p\right)-{\hat{y}}_{k,l}\left(s,s\right)\right]}^{2}\right\}.} \end{array}$$

Moreover, the least square plans with size of *s* × *s* were used to fit the *s*th segment of local trends in images. Then, the *s*th trend can be removed from the integrated image to derive the *s*th fluctuation. DFA scaling exponent is defined as the power-law correlation between the using scales and the derived fluctuations. Finally, the DFA scaling exponent **α** by the straight line of log⁡⁡(*F*(*s*)) versus log⁡⁡(*s*) plot is obtained.

### 2.3. Statistical Analysis

Values were expressed as means ± SD. Data were analyzed by one-way analysis of variance (ANOVA) (SigmaStat statistical software, Jandel Scientific, San Rafael, CA). The Tukey test was conducted for multiple comparisons when the null hypothesis was not applicable for the same group. It was also used for all pairwise comparisons of the mean responses to the different treatment groups. Differences were considered significant at a value of *P* < 0.05.

## 3. Results and Discussion

### 3.1. Simulation

**Figure 3:**
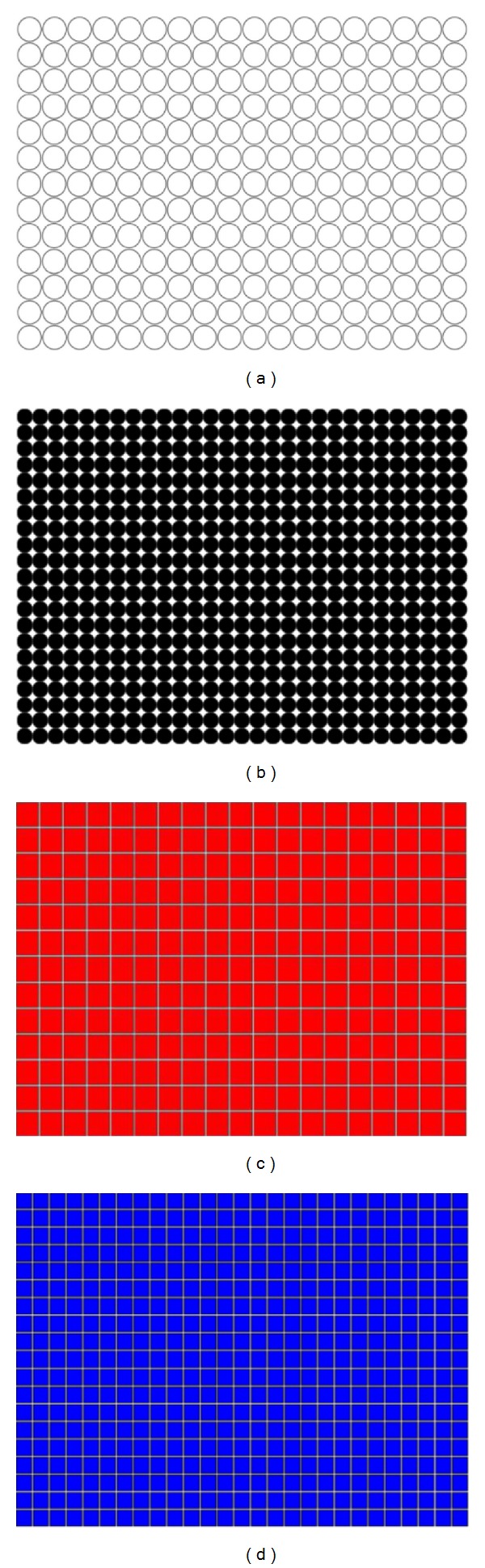
Simulation images. (a) White large circle, (b) black small circle, (c) red large square, and (d) blue small square, which image is 4080 × 3072 pixels. The shape of image has circle and square, two types. Every type's color has white, black, red, green, blue, and mix color, six modes, and size has large and small, two categories.

**Figure 4:**
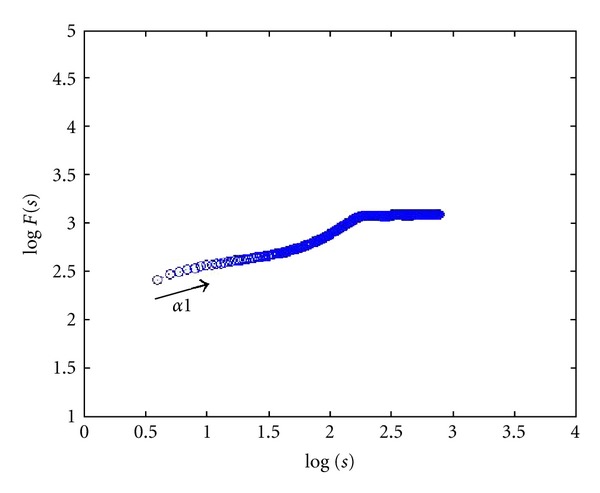
Red small circle image plot of log⁡⁡(*F*(*s*)) versus log⁡⁡(*s*) using DFA analysis method.

24 images (4080 × 3072 pixels, same as lymph section images scale) were used to check the proposed two-dimensional analysis DFA method for image analysis. Different shape, color, and size images are used. Some images of simulation are shown in Figure 3. The image shape has circle and square, two types, which color has white, black, red, green, blue, and mix color, six modes, and size has large and small, two categories. The scaling exponent **α** can be estimated by a linear fit on the log-log plot of *F*(*s*) versus *s*. The monodimensional DFA method has three types **α** value. It represents the correlation properties of the statistically self-similarity. Reflecting on two-dimensional DFA method, the global scaling exponent **α** value was calculated within the range of *s* between *s* = 4 and *s* = min⁡⁡(*M*/4, *N*/4), the short-term correlation exponent **α**1 was calculated within the range between *s* = 4 and *s* = 11 [7, 39, 40], and the long-term correlation exponent **α**2 was calculated within the range of *s* between *s* = 12 and *s* = min⁡⁡(*M*/4, *N*/4). Plotted log⁡⁡*F*(*s*) versus log⁡⁡*s* picture by the DFA analysis method of red small circle image was shown in Figure 4. End of the plotted curve is displayed nearly the horizon line. This trend is similar as other 23 simulation images. Because this curve trend, not displaying the line slope style, is unable to calculate the **α** and **α**2 values, we selected **α**1 to calculate total 24 simulation images and compared between the different shape, color, and size as shown in Table 2. The results show that different shapes, colors, and size images have different **α**1 values. The 24 simulation images show three cases. First case is similar shape with same color which is all the large size **α**1 value bigger than the small size. The small size shape means the numbers are more, and the shape area and length have larger values and complexity which are the same **α** exponent roughness concept of monodimensional DFA method from Peng et al. [6]. It is found that complex images have lower value of **α**1. Second case is similar size (i.e., the square diagonal length equal circle diameter) and color situation where the square shape **α**1 value is greater than or equal (only white large image) the circle shape **α**1 value. This observation corresponds the larger area image (i.e., circle area) and has lower **α**1 value as initially observed. Third case is similar shape and size situation where **α**1 values of different colors almost have no significant differences. This means the image color influence is very low when using 2D DFA method to analysis image characteristic. Therefore, when clinical images are influenced by light, the resulting images which are different in colors and cause a change in the characteristic value can be reduced. In summary, the index of the **α**1 value has distinctive capability and consistency in image analysis.

### 3.2. Healthy Cell, BL and DLBCL Lymphoma Image Calculated Results

**Figure 5:**
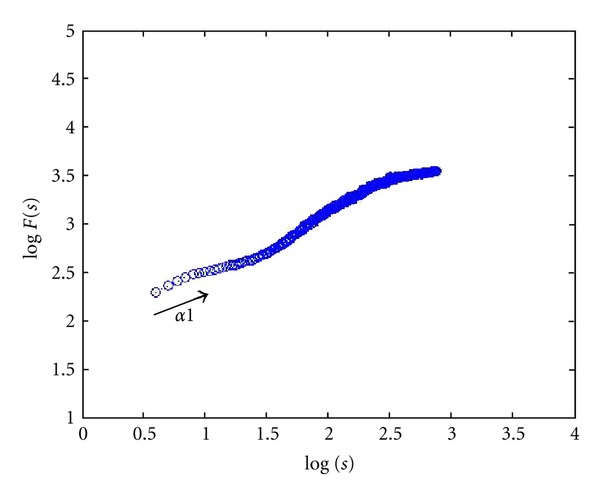
Healthy cell image plot of log⁡⁡(*F*(*s*)) versus log⁡⁡(*s*) using DFA analysis method.

**Figure 6:**
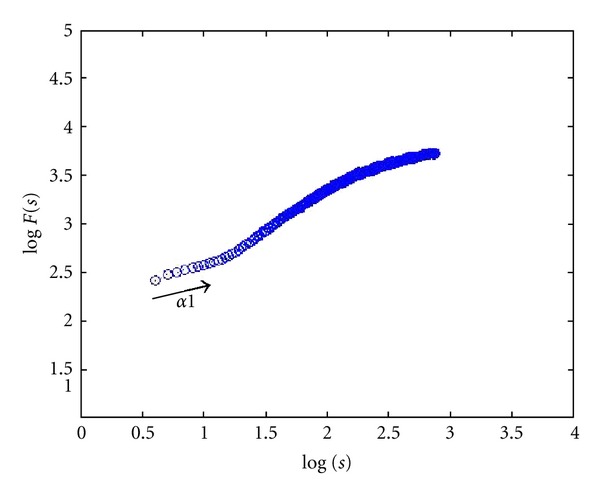
BL image (more than five cytogenetic changes) plot of log⁡⁡(*F*(*s*)) versus log⁡⁡(*s*) using DFA analysis method.

Figure 5 shows a healthy cell image plotted using log⁡⁡(*F*(*s*)) versus log⁡⁡(*s*) picture based on the DFA analysis method.  Figure 6 shows a BL image of group B. All the simulation images show that the lymphoma images have similar curve trend. Therefore the DFA method was used to calculate **α**1 value for a healthy cell image for a total of 38 lymphoma images classified into three groups. The **α**1 value for healthy cell of Figure 2(a) is 0.50 which is due to the clarity of healthy cell images. The mean values of the short-term correlation exponent **α**1 of groups A, B, and C are 0.370 ± 0.033, 0.382 ± 0.022, and 0.435 ± 0.053, respectively, as shown in Table 3. It is observed that the healthy cell **α**1 value was different from lymphoma cells. Furthermore, the **α**1 value of BL image (both BLs) was significantly lower (*P* < 0.05) than DLBCL image (including groups A and C, groups B and C) and has no difference between both BLs (groups A and B). Hence, it could be concluded that **α**1 values based on DFA statistics concept could clearly distinguish pathologic states between BL and DLBCL images.

## 4. Conclusions

DFA method was utilized to measure the scale invariant behavior that evaluates trends in the presence or absence of fractal correlation properties of time series data [8, 9]. In this study, 2D DFA method was derived and used to explore the trend of fractal images where the **α**1 value can be easily identified.

In this paper, the DFA method has been applied to image analysis. The two-dimensional matrix algorithm of DFA, both integration and detrending processes, was used for time series data field. The DFA method has been used to investigate the characteristic of different type of simulated and lymphoma image. The lymphoma images test results show that the short-term correlation exponent **α**1 value of DFA obtained from BL and DLBCL have statistical significant difference. This result is very encouraging, which **α**1 value could be an index, to help the doctor for distinguishing between BL and DLBCL.

However, the authors had been testing the matrix performance of a two-dimensional image as in equation (1) of [26]. The 2D DFA results calculation of the matrix performance are very time consuming. For example, one image (4080 × 3072 pixels) takes over three days. This is disadvantageous for the real-time requirements. Therefore, the 1D DFA concept is being used to solve the aforementioned image's problem. We assume the lymphoma cell shape, color, and size were influenced by the row and column cells before it. By this way, the verification results of calculation time were great reduced for the 2D DFA, about 3 hours for one image. Fortunately, statistical analysis of calculation results can distinguish between BL and DLBCL lymphoma. However, groups A and B in BL still cannot be classified in this study. Therefore, further investigations are needed to improve the sensitivity and specificity of classification. Results of this study can be compared to 2D DFA algorithm in [26] or 2D-DMA algorithm in [41, 42] to investigate how widely this method can be applied in clinical analysis.

## Figures and Tables

**Table 1:** The groups of Burkitt lymphoma (BL) and diffuse large B cell lymphoma (DLBCL) section images and case number.

Groups			Case number
A	Burkitt lymphoma (BL)	One to five cytogenetic changes	18
B		More than five cytogenetic changes	10
C	Diffuse large B cell lymphoma (DLBCL)		10

**Table 2:** The DFA **α**1 value of 24 simulation images.

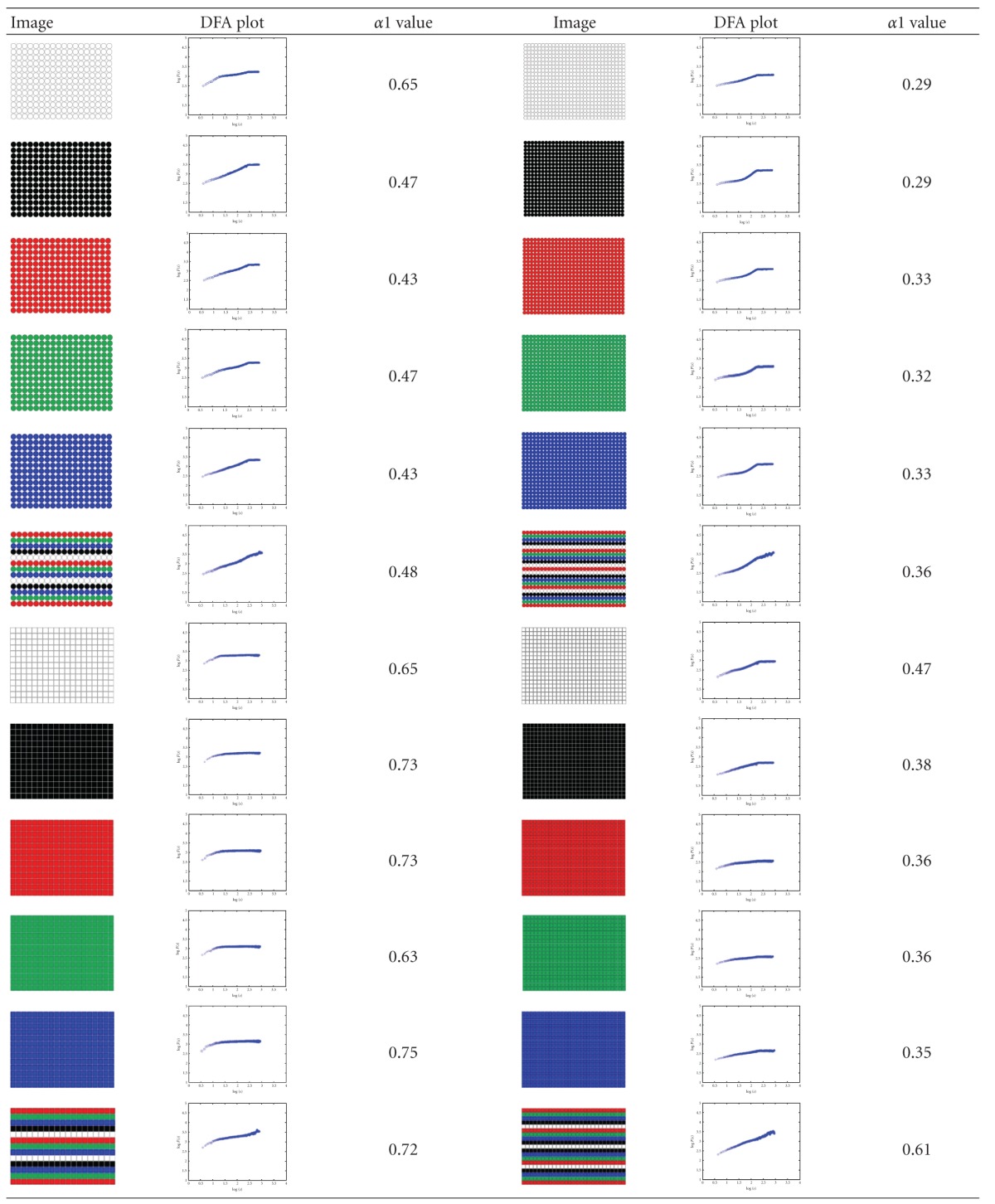

**Table 3:** The DFA *α*1 value of Burkitt and DLBCL images.

Classification	Group A	Group B	Group C	*P* value
*α*1 value	0.370 ± 0.033^a^	0.382 ± 0.022^b^	0.435 ± 0.053	<0.001

Values are expressed as mean ± standard deviation.

*P* < 0.05 was considered statistically significant difference using the ANOVA method.

^a^ *P* < 0.05 for group A versus group C comparison using the Tukey test.

^b^ *P* < 0.05 for group B versus group C comparison using the Tukey test.
